# Significance of Nuclear Morphometry in Breast Lesions: A Cross-Sectional Study

**DOI:** 10.7759/cureus.39378

**Published:** 2023-05-23

**Authors:** Ankita Girdhar, Kalyani Raju, Sreeramulu P N

**Affiliations:** 1 Department of Pathology, Sri Devaraj Urs Medical College, Sri Devaraj Urs Academy of Higher Education and Research, Kolar. Karnataka. India., Kolar, IND; 2 Department of Pathology, Sri Devaraj Urs Medical College, Sri Devaraj Urs Academy of Higher Education and Research, Kolar, IND; 3 Department of General Surgery, Sri Devaraj Urs Medical College, Sri Devaraj Urs Academy of Higher Education and Research, Kolar, IND

**Keywords:** nuclear morphometry., fnac of breast, cytology of breast, breast neoplasm, breast lesions

## Abstract

Background

Fine-needle aspiration cytology (FNAC) is one of the reliable methods in diagnosing breast cancers. Morphometric studies are done in benign and malignant neoplasms of various organs by using software, which measures cellular, cytoplasmic, and nuclear parameters. Nuclear parameters define the behavior of the neoplasm. This study aims to evaluate nuclear morphometry parameters in aspirated smears of breast lesions and determine the association between cytological findings with nuclear morphometry parameters.

Methodology

It’s a retrospective cytology study from July 2020 to June 2022 conducted at a tertiary health care center in Kolar, Karnataka, India. The FNAC smears of breast mass were analyzed cytologically and were subjected to nuclear morphometry study. Nuclear parameters such as nuclear area, nuclear perimeter, nuclear Feret diameter, minimum Feret, and shape factor were captured in Zen software (Zeiss, Oberkochen, Germany) and ImageJ software (National Institutes of Health, Bethesda, MD, USA; Laboratory for Optical and Computational Instrumentation [LOCI], University of Wisconsin-Madison, Madison, WI, USA). The association between nuclear morphometric findings and cytological findings was noted. A descriptive statistical analysis was done.

Results

Sixty cases of mass in the breast were considered for the study of which 37 cases were benign and 23 were malignant. Nuclear morphometry parameters such as nuclear area, nuclear perimeter, nuclear Feret diameter, minimum Feret, and shape factor for benign breast lesions were 25.16 ± 3.2 µm^2^, 21.58 ± 1.89 µm, 6.5 ± 0.94 µm, 4.87 ± 0.50 µm, and 0.92 ± 0.02, respectively, and for malignant breast cases were 46.57 ± 12.24 µm^2^, 27.53 ± 3.26 µm, 10.08 ± 1.18 µm, 6.49 ± 0.88 µm, and 0.93 ± 0.01, respectively. The association of all nuclear parameters between benign and malignant lesions was statistically significant (*P* = 0.001).

Conclusions

Nuclear morphometric study in breast lesions is a concept that supplements FNAC findings in differentiating benign from malignant lesions.

## Introduction

Globally, breast cancer (BC) is one of the commonest cancer among women. Over the past decades, BC incidence is increasing and has become the number one cancer globally. According to GLOBOCAN 2020, the incidence of BC is 2.26 million cases (11.7%) worldwide [1]. Among Indians, cases of BC are at the top of the rank list. According to statistics of BC in India for the year 2018, newly detected BC cases in India were 162,468 (27.7%) and mortality was 87,090 (23.5%). Approximately, one in four women were newly diagnosed and died due to BC in India [2]. The proportion of BC in Bangalore is 34.4% [3]. The prevalence of BC in Kolar, Karnataka, reported is 6.4% of all female cancers [4].

For cytological evaluation of BC, fine-needle aspiration cytology (FNAC) is one of the commonest routinely used methods, which is a simple, quick, and inexpensive method. Of late, a core needle biopsy is emerging as the ideal method. However, FNAC is one of the reliable methods for diagnosing BC. It helps in diagnosing patients preoperatively [5]. In the literature, the accuracy rate for FNAC in breast lesions ranges from 95.8% to 97.87% [6].

When a disease transforms from a benign lesion to a malignancy lesion, there is variation in nuclear parameters. To diagnose malignancy, variation in nuclear structure is the most important morphological feature [5]. The grading of cytological diagnosis of breast aspirate is done by various methods such as Robinson’s grading, grading as per IAC protocol, etc. [7,8]. Direct microscopy of FNAC smears evaluates only size and other morphological features, while computer-based images evaluate the nuclear size, shape, texture, and density parameters. Nuclear morphometry is a method of analyzing images by measuring various nuclear parameters by using software that measures cellular, cytoplasmic, and nuclear parameters [5].

Morphometric studies are done in benign and malignant neoplasms of various organs. In this study, nuclear morphometry study was done on FNAC breast smears. The FNAC smears of breast neoplasms were analyzed cytologically. Nuclear morphometry parameters were studied in aspirated smears. The association between cytological findings with nuclear morphometry was evaluated by using Zen software (Zeiss, Oberkochen, Germany).

## Materials and methods

This study is a laboratory observational retrospective cross-section study from July 2020 to June 2022 at the Cytology Section, Department of Pathology, in a tertiary healthcare hospital attached to Sri Devaraj Urs Medical College, Kolar, Karnataka. Institutional ethical clearance was taken before the start of the study (DMC/KLR/IEC/352/2022-23, on September 06, 2022). Cases with mass in the breast were considered for the study.

Inclusion criteria were all the FNAC smears of breast mass, with smears showing clarity in nuclear and cytoplasmic features. Exclusion criteria were all the smears with inadequate staining, showing overlapping of nuclei, with abundant necrotic and degenerative material, abundant inflammatory cell infiltrate, borderline proliferative breast lesions, mucous and blood, unlabeled smears, and broken slides. Of the total of 72 cases, 60 (83.3%) were included (37 benign [fibroadenoma] and 23 malignant [ductal carcinoma]) and 12 (16.6%) were excluded (7 marked necrosis and 5 scant cellularities).

FNAC smears stained with Papanicolaou (PAP) stain were considered for the study. The cytological evaluation was based on cellularity, cellular morphology, abnormal chromatin pattern, nucleoli size, and mitotic activity. As per the cytomorphology, the cases were classified as benign and malignant lesions (Tables 1-2).

**Table 1:** Cytomorphology of benign breast lesions.

S. no.	UHID	Cellularity	Morphology	Nuclear atypia	Nucleoli	Mitotic activity
1	882814	Low	Benign	Absent	Indistinct	Absent
2	883719	Low	Benign	Absent	Indistinct	Absent
3	891520	Low	Benign	Absent	Indistinct	Absent
4	895395	Low	Benign	Absent	Indistinct	Absent
5	898269	Low	Benign	Absent	Indistinct	Absent
6	902844	Low	Benign	Absent	Indistinct	Absent
7	903446	Low	Benign	Absent	Indistinct	Absent
8	908129	Low	Benign	Absent	Indistinct	Absent
9	926183	Low	Benign	Absent	Indistinct	Absent
10	925864	Low	Benign	Absent	Indistinct	Absent
11	933344	Low	Benign	Absent	Indistinct	Absent
12	936509	Low	Benign	Absent	Indistinct	Absent
13	936742	Low	Benign	Absent	Indistinct	Absent
14	916741	Low	Benign	Absent	Indistinct	Absent
15	937846	Low	Benign	Absent	Indistinct	Absent
16	950450	Low	Benign	Absent	Indistinct	Absent
17	39217	Low	Benign	Absent	Indistinct	Absent
18	41060	Low	Benign	Absent	Indistinct	Absent
19	49007	Low	Benign	Absent	Indistinct	Absent
20	52136	Low	Benign	Absent	Indistinct	Absent
21	50850	Low	Benign	Absent	Indistinct	Absent
22	55115	Low	Benign	Absent	Indistinct	Absent
23	55477	Low	Benign	Absent	Indistinct	Absent
24	52135	Low	Benign	Absent	Indistinct	Absent
25	56218	Low	Benign	Absent	Indistinct	Absent
27	57045	Low	Benign	Absent	Indistinct	Absent
28	60877	Low	Benign	Absent	Indistinct	Absent
29	53958	Low	Benign	Absent	Indistinct	Absent
30	62541	Low	Benign	Absent	Indistinct	Absent
31	60827	Low	Benign	Absent	Indistinct	Absent
32	70343	Low	Benign	Absent	Indistinct	Absent
33	70874	Low	Benign	Absent	Indistinct	Absent
34	71915	Low	Benign	Absent	Indistinct	Absent
35	75010	Low	Benign	Absent	Indistinct	Absent
36	77633	Low	Benign	Absent	Indistinct	Absent
37	84721	Low	Benign	Absent	Indistinct	Absent

**Table 2:** Cytomorphology of malignant breast lesions.

S. no.	UHID	Cellularity	Morphology	Nuclear atypia	Nucleoli	Mitotic activity
1	887897	High	Malignant	Present	Present	Present
2	893855	High	Malignant	Present	Present	Present
3	903629	High	Malignant	Present	Present	Present
4	922046	High	Malignant	Present	Present	Present
5	923833	High	Malignant	Present	Present	Present
6	926208	High	Malignant	Present	Present	Present
7	930986	High	Malignant	Present	Present	Present
8	929311	High	Malignant	Present	Present	Present
9	946403	High	Malignant	Present	Present	Present
10	952410	High	Malignant	Present	Present	Present
11	40374	High	Malignant	Present	Present	Present
12	50229	High	Malignant	Present	Present	Present
13	53456	High	Malignant	Present	Present	Present
14	65320	High	Malignant	Present	Present	Present
15	68494	High	Malignant	Present	Present	Present
16	75084	High	Malignant	Present	Present	Present
17	79347	High	Malignant	Present	Present	Present
18	19020	High	Malignant	Present	Present	Present
19	82486	High	Malignant	Present	Present	Present
20	87083	High	Malignant	Present	Present	Present
21	94019	High	Malignant	Present	Present	Present
22	73696	High	Malignant	Present	Present	Present
23	15416	High	Malignant	Present	Present	Present

Histopathology diagnosis was considered the gold standard in all the cases (Table 3). 

**Table 3:** Cytohistopathological correlation.

		Frequency	Histopathology	Cytohistpathology correlation
Benign (*n *= 37)	Fibroadenoma breast	25	9	9
	Fibrocystic disease	12		
Malignant (*n *= 23)	Ductal carcinoma	23		
Total		60	9	9

**Figure 1:**
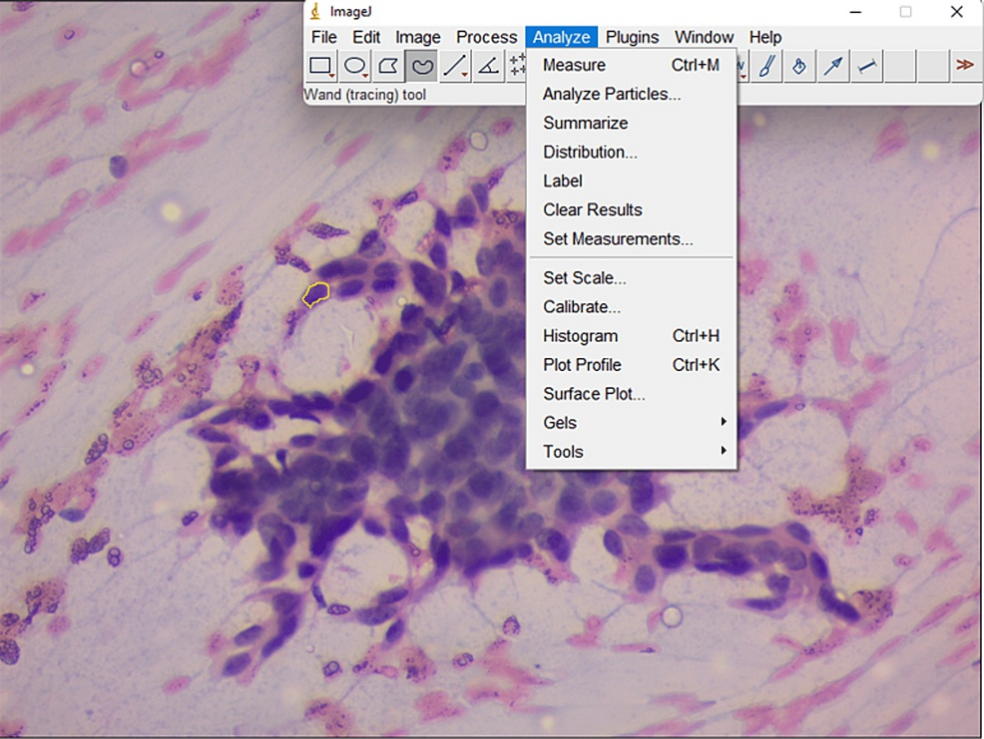
Microphotograph of cytology of benign breast cancer with software for morphometry (PAP 400×). PAP, Papanicolaou

**Figure 2:**
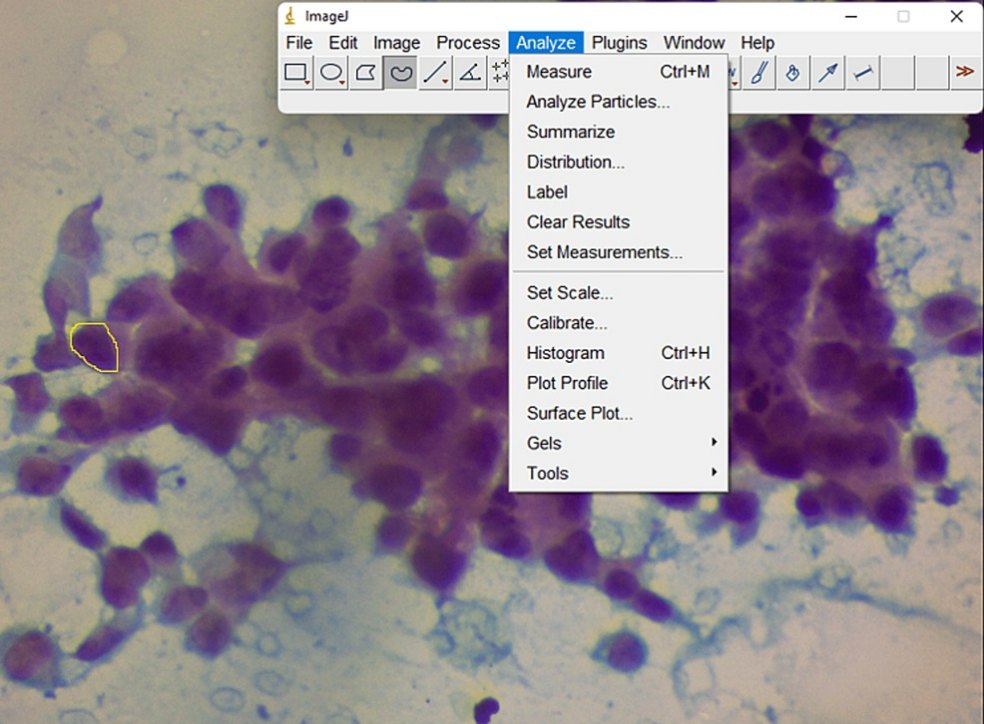
Microphotograph of cytology of malignant breast cancer with software for morphometry (PAP 400×). PAP, Papanicolaou

Next, nuclear morphometry analysis was done. Fifty nuclei of the ductal cells, which were not overlapping in the FNAC smear, were considered. Images were captured at 400× magnification using Zen software and saved in the computer in JPEG format. Background correction was done for uniformity of image intensity. The saved images were further processed with ImageJ software (National Institutes of Health, Bethesda, MD, USA; Laboratory for Optical and Computational Instrumentation [LOCI], University of Wisconsin-Madison, Madison, WI, USA; Figures 1-2). Calibration was performed manually in Zen software using a built-in scale. Then nuclear parameter values was derived from ImageJ software. The software was used before for the morphometry study of lesions in other organs. A distance was assigned to one pixel (by calibration), and automatic measurement was done by comparing objects of different images. Measurement was done in pixels. Nuclear morphometric parameters were analyzed by nuclear size, nuclear shape, and nuclear texture, and nuclear density parameters by nuclear area, nuclear perimeter, nuclear Feret, minimum Feret, and shape factor (Table 4) [5,6]. The parameters were compared between benign and malignant groups. The association between nuclear morphometric findings and cytological findings was assessed.

**Table 4:** Definition of nuclear parameters in morphometry analysis.

Nuclear morphometric parameters used in this study	Definition
Nuclear area	Area of the nucleus
Nuclear perimeter	Total boundary measurement of nucleus
Nuclear Feret	Maximum value of the set of Feret diameter
Minimum Feret	Minimum value of the set of Feret diameter
Shape factor	Measures roughness of the nucleus

The data were entered in a Microsoft Excel sheet, and statistical analysis was done by using IBM SPSS Statistics for Windows, Version 22.0 (IBM Corp., Armonk, NY, USA). Quantitative data were expressed as mean and standard deviation and were analyzed by Student's *t-test*. Qualitative data (cytological diagnosis) were expressed as frequency and proportion and were analyzed by the chi-square test. *P-*value <0.05 was considered statistically significant.

## Results

The study sample size was 60, of which 37 (61.6%) were benign and 23 (38.3%) were malignant. It was a cross-sectional study conducted from July 2020 to June 2022. Age distribution for benign and malignant breast lesions was between 21 and 75 years and 40 and 65 years, respectively. The mean age along with the standard deviation values of benign and malignant lesions was 40.43 ± 13.46 and 49.91 ± 11.75, respectively. All cases were females. Out of 60 cases, the majority were premenopausal females (*n *= 33) and the remaining were postmenopausal females (*n *= 27). Among the 37 benign breast lesions, 23 (64.86%) cases were fibroadenoma and 14 (35.14%) were fibrocystic disease. All 23 (100%) malignant breast lesions were invasive ductal carcinoma (Table 5).

**Table 5:** Cytomorphological distribution of benign and malignant lesions in this study.

Benign breast lesion (*n *= 37)	Frequency	Percentage (%)
Fibroadenoma	24	64.86
Fibrocystic disease breast	13	35.14
Total	37	100
Malignant breast lesion (*n *= 23)		
Ductal carcinoma	23	100
Total	23	100

Nuclear morphometry parameters such as nuclear area, nuclear perimeter, nuclear Feret diameter, minimum Feret, and shape factor for benign breast lesions were 25.16 ± 3.2 µm^2^, 21.58 ± 1.89 µm, 6.5 ± 0.94 µm, 4.87 ± 0.50 µm, and 0.92 ± 0.02, respectively, and for malignant breast cases were 46.57± 12.24 µm2, 27.53 ± 3.26 µm, 10.08 ± 1.18 µm, 6.49 ± 0.88 µm, and 0.93 ± 0.01, respectively. All the nuclear morphometric parameters were higher in malignant breast cases compared to benign breast cases. The *P*-values of the nuclear parameters such as nuclear area (*P *= 0.001), nuclear perimeter (*P *= 0.001), nuclear Feret (*P *= 0.001), minimum Feret (*P *= 0.05), and shape factor (*P *= 0.5) between benign and malignant cases were statistically significant. Nuclear parameters mean values in benign and malignant diseases along with standard deviation and *P*-value are shown in Table 6.

**Table 6:** Mean values of the nuclear morphometry parameters in benign and malignant cases in this study.

Nuclear parameters	Benign breast disease	Malignant breast disease	*P*-value
Nuclear area	25.16 ± 3.2 µm^2^	46.57 ± 12.24 µm^2^	0.001
Nuclear perimeter	21.58 ± 1.89 µm	27.53 ± 3.26 µm	0.001
Nuclear Feret	6.5 ± 0.94 µm	10.08 ± 1.18 µm	0.001
Minimum Feret	4.87 ± 0.50 µm	6.49 ± 0.88 µm	0.05
Shape factor	0.92 ± 0.02	0.93 ± 0.01	0.5

## Discussion

BC is one of the leading causes of death in India. There is an alarming rise in the frequency of BC worldwide, and it is one of the most common cancers in females worldwide [3]. The incidence rate of BC in India is 25.8/100,000 population, and the mortality rate is 12.7/100,000 [9]. Hence, screening (both imaging and pathology) of healthy women is a necessary modality. Various diagnostic modalities such as FNAC, histopathology biopsies, etc., help in reaching specific diagnoses [7,10].

FNAC is an easy, simple, cost-effective, and reliable method for diagnosing BC and helps to diagnose preoperatively. But there are certain pitfalls associated with FNAC procedures which include interobserver variability for parameters such as cellularity, nuclear morphology, and overlapping of nuclei, which come under come under the gray zone [6,11,12]. Hence, morphometric studies with digital software can be used to overcome the related pitfalls. This method is more precise in measuring various nuclear parameters than conventional FNAC [12,13,14,15]. The various digital techniques are more accurate and give objective diagnoses, which helps to predict the prognosis of the disease [7,10].

According to a study by Kalhan et al., morphometric studies were conducted on cytologically confirmed breast carcinoma cases using various nuclear parameters, and the study helped in prognostication in BC (Table 7) [8].

**Table 7:** Mean values of nuclear morphometry in benign and malignant lesions in various studies compared to this study.

Parameters		This study	Kashyap et al. (2017) [5]	Kalhan et al. (2021) [8]	Niranjan Pandian et al. (2021) [9]	Krishnappa et al. (2018) [13]
Sample size	Total	60	122	82	2,000	49
	Benign	37	58	53	1,000	25
	Malignant	23	64	29	1,000	24
Nuclear area (µm^2^)	Benign	25.16 ± 3.2	25.49 ± 3.88	78.21 ± 12.40	16.67 ± 5.41	107.51 ± 35.62
	Malignant	46.57 ± 12.24	51.43 ± 20.47	93.12 ± 13.85	39.63 ± 22.41	298.86 ± 112.7
Nuclear perimeter (µm)	Benign	21.58 ± 1.89	18.39 ± 1.49	31.08 ± 4.24	15.79 ± 2.90	38.32 ± 6.29
	Malignant	27.53 ± 3.26	25.69 ± 4.99	36.81 ± 3.77	23.62 ± 6.93	63.62 ± 14.74
Nuclear Feret (µm)	Benign	6.5 ± 0.94	6.61 ± 0.57	-	5.71 ± 1.04	-
	Malignant	10.08 ± 1.18	9.26 ± 1.81	-	8.38 ± 2.15	-
Minimum Feret (µm)	Benign	4.87 ± 0.50	5.11 ± 0.41	-	-	-
	Malignant	6.49 ± 0.88	7.09 ± 1.29	-	-	-
Shape factor	Benign	0.92 ± 0.02	0.95 ± 0.02	1.05 ± 0.01	-	0.898 ± 0.029
	Malignant	0.93 ± 0.01	0.94 ± 0.01	1.09 ± 0.04	-	0.93 ± 0.28
*P*-value between benign and malignant lesions		<0.001	<0.0001	<0.05.	≤0.05.	<0.05

Another study by Niranjan Pandian et al. showed that the morphometric studies conducted with ImageJ software helped in differentiating between benign and malignant breast lesions. In this study, a total of six nuclear parameters were considered - nuclear area, nuclear perimeter, nuclear diameter, density parameters (integrated and raw) measuring nuclear chromasia, and axis ratio (shape parameter), which showed a *P-*value <0.05, indicating statistical significance (Table 7) [9].

In a study by Krishnappa et al., the cytomorphology of FNAC breast aspirate smears was correlated with the nuclear morphometric study results. Aspirates from malignant breast aspirates were graded according to *Robinson’s cytological grading* as grade I (scores 1-11), grade II (scores 12-14), and grade III (scores 15-18) and correlated with nuclear morphometry parameters. The nuclear morphometry parameters showed an increase in values from grade I to grade III malignant breast cases. Hence, the study concluded that nuclear morphometry was an effective tool in diagnosing fine-needle aspirates of breast masses and thus helped in differentiating benign from malignant breast masses (Table 7) [13].

According to Kashyap et al., 50 benign breast disease (BBD) and 64 carcinomas cases were considered for their study. All nuclear parameters such as the mean nuclear area, equivalent diameter, minimum Feret, maximum Ferret, and perimeter between benign and malignant cases were found to be statistically significant in differentiating between benign and malignant cases, with *P* < 0.001. The wide variation in the different studies was due to different software used in different studies (Table 7) [5].

In this study, the cytological diagnosis was done on FNAC of breast aspirate smears. Later, nuclear morphometry studies were conducted using ImageJ software. The nuclear parameters considered were the nuclear area, nuclear perimeter, nuclear Feret, minimum Feret, and shape factor. There was an increase in the mean nuclear parameter values in malignant breast cases compared to benign cases. Hence, nuclear morphometry studies aided in categorizing benign and malignant breast cases. The diagnosis and differentiation of benign and malignant lesions can be done by morphometry and can be used in screening FNAC smears, especially in primary healthcare centers where pathologists are not available.

The limitations of this study were as follows: it was a unicentric and time-consuming study. The laboratory personnel had to be trained and skilled in using the software. However, nuclear morphometry parameters showed a statistically significant association between benign and malignant breast neoplasms. The method should be standardized for routine use in primary healthcare centers.

The concept can be taken forward for automation in cytology and used for screening breast aspirates in primary healthcare centers where there is less availability of cytologists.

## Conclusions

In this study, there was a statistically significant association of nuclear morphometry parameters between benign and malignant lesions of the breast. This information can be used for automation in breast aspirate cytology, screening breast aspirate smears, and management of breast lesions.
